# A new strategy for CAR-T therapy in solid tumors: IL-15-autocrine signaling augments tumor stroma depletion and promotes a T_SCM_ subset in the TME

**DOI:** 10.1038/s41419-025-08405-2

**Published:** 2025-12-27

**Authors:** Yanyang Pang, Leiyuan Chen, Qinghui Sun, Na He, Zhiheng Lai, Xi Wang, Zhihao Xie, Wenli Yang, Wu Wang

**Affiliations:** 1 School of Traditional Chinese Medicine, Hainan Medical University, Haikou, China; 2 Public Research Center of Hainan Medical University, Hainan Medical University, Haikou, China; 3 Key Laboratory of Emergency and Trauma of Ministry of Education, Key Laboratory of Tropical Cardiovascular Diseases Research of Hainan Province, Engineering Research Center for Hainan Biological Sample Resources of Major Diseases, The First Affiliated Hospital of Hainan Medical University, Haikou, China; 4 NHC Key Laboratory of Tropical Disease Control, School of Life Sciences and Medical Technology, Hainan Medical University, Haikou, China; 5 Department of Anorectal surgery, Guangdong Provincial Hospital of Chinese Medicine, Hainan Hospital, Guangzhou University of Chinese Medicine, Guangzhou, China; 6 Department of Anesthesiology, Haikou Third People’s Hospital, Haikou, China; 7 Department of Anatomy, Zunyi Medical University, Zunyi, China

**Keywords:** Cancer immunotherapy, Targeted therapies

## Abstract

Although chimeric antigen receptor (CAR)-T cell therapy has achieved remarkable therapeutic effects in treating hematologic cancers, its effectiveness in solid tumors remains significantly restricted. the primary reason is the immunosuppression mediated by the tumor microenvironment (TME), which leads to rapid exhaustion of infiltrating CAR-T cells. To enhance CAR-T cell efficacy against solid tumors, we pursued improvements in two aspects. First, we constructed fibroblast activation protein (FAP)-directed CAR-T cells to enhance their anti-CAF capability within the TME, thereby alleviating the immunosuppressive barrier. Second, we utilized IL-15, an efficient activator of CAR-T cells that inhibits activation-induced cell death, restores effector functions, and increases the proportion of the T stem cell memory (T_SCM_) subpopulation. In this study, we report the generation of FAP/IL-15 CAR-T cells, which target FAP and autonomously synthesize and secrete IL-15. Our data demonstrate that treatment with FAP/IL-15 CAR-T cells exhibited stronger activation characteristics in a FAP antigen-dependent manner, selectively targeting CAFs within the solid TME. Moreover, endogenous IL-15 secretion enabled CAR-T cells to adopt a T_SCM_-like phenotype with enhanced memory characteristics, thus improving cell survival, proliferation, activation, and therapeutic efficacy against solid tumors.

## Introduction

In recent years, CAR-T cell-based immunotherapy has emerged as an innovative and effective therapeutic strategy for tumor eradication. Through genetic modification, T cells are equipped with engineered CAR molecules that confer specificity toward selected tumor-associated antigens. Unlike traditional adoptive immune cell therapies, CAR-T cells recognize tumor antigens in an MHC-independent manner, significantly enhancing their ability to transmit activation signals and initiate immune responses [1]. Numerous clinical trials have demonstrated that CAR-T cell therapy promotes substantial clinical responses in hematological malignancies, including acute lymphoblastic leukemia (ALL), chronic lymphocytic leukemia (CLL), multiple myeloma, and non-Hodgkin lymphoma [2]. Currently, the FDA has approved multiple CAR-T cell products targeting CD19 or B-cell maturation antigen (BCMA) for clinical trials and for treating relapsed/refractory B-cell malignancies (r/r B-ALL) and multiple myeloma [3]. CD19-targeted CAR-T cells achieve initial complete response rates approaching 85% in ALL patients and nearly 100% in r/r B-ALL cases [4, 5]. These therapies have demonstrated significant clinical promise and are now approved as second-line treatments following chemotherapy failure in r/r B-ALL patients [6]. Despite breakthroughs in B-cell malignancies, CAR-T therapy has shown limited efficacy against solid tumors and faces substantial challenges. The highly immunosuppressive TME in solid tumors leads to rapid CAR-T cell exhaustion upon infiltration, severely compromising their antitumor activity [7]. Additionally, factors such as off-target toxicity, cytokine release syndrome, and cytopenia-related adverse events associated with CAR-T cell side effects pose substantial challenges to clinical application [8].

The TME results from complex interactions between tumor cells, fibroblasts, immune cells, endothelial cells, extracellular matrix (ECM), microvessels, and infiltrating biomolecules [9]. Functioning as both a growth-promoting niche and a protective shield, the TME forms physical barriers composed of stromal cells and ECM. These barriers impair CAR-T cell infiltration, impede proper trafficking to tumor regions, and limit long-term persistence, collectively contributing to CAR-T treatment failure in solid tumors [10]. Current research explores incorporating targets associated with TME elements (e.g., fibroblasts, immune cells, vasculature, ECM) into CAR designs, termed “TME-targeting CAR-T” or “stroma-targeting CAR-T” [11, 12]. FAP, a type II transmembrane serine protease glycoprotein, is prominently overexpressed on cancer-associated fibroblasts (CAFs) within the tumor-associated stroma across multiple human epithelial malignancies [13]. Although CAR-T therapies targeting FAP-expressing stromal components have shown promising results [14, 15], challenges remain that limit their widespread adoption and clinical efficacy. In preclinical studies, a primary challenge in optimizing FAP-targeting CAR constructs is enhancing the efficiency and precision of effector T cells within solid tumors. Nevertheless, this CAF-directed strategy provides a viable foundation for developing effective TME-targeting CAR-T therapies.

Additionally, efforts must address the limitations imposed by TME-mediated immunosuppression on CAR-T cell survival, including improvements in intratumoral infiltration capability and lifespan. Although current evidence suggests that combining CAR-T cells with immune checkpoint inhibitors (e.g., PD-1, PD-L1, CTLA-4) creates a synergistic effect that significantly enhances intratumoral function and survival compared to monotherapies, issues remain, such as low response rates and drug resistance. Therefore, discovering new activation targets for CAR-T cells remains urgent [16, 17]. Interleukin-15 (IL-15), a pivotal cytokine regulating adaptive and innate immunity, plays a critical role in promoting T-cell survival and proliferation [18]. Studies demonstrate that IL-15 inhibits activation-induced CAR-T cell death, restores effector function, and significantly enhances anti-tumor efficacy in vivo [19]. Notably, the stem-like central memory T cell (T_SCM_) subpopulation typically represents only a small fraction of CAR-T cells applied in solid tumors due to suppression by the TME [20, 21]. T_SCM_ cells represent the earliest differentiation stage of antigen-stimulated T cells. As progenitors of all memory T cells, they exhibit stem cell-like properties, including potent self-renewal capacity [22, 23]. Exogenous IL-15 stimulation enables CAR-T cells to adopt a T_SCM_-like phenotype with enhanced memory characteristics, thus improving their effectiveness against solid tumors [24]. CAR-T cell therapies employing IL-15, either via ex vivo cell expansion supplemented with IL-15 or through combination strategies involving other therapeutic modalities, hold considerable potential for future clinical translation.

**Fig. 1:**
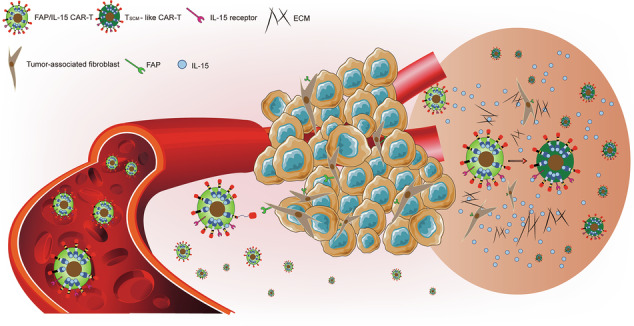
Therapy strategy with FAP/IL-15 CAR-T cells treatment. By targeting FAP, activated FAP/IL-15 CAR-T cells can autonomously synthesize and secrete IL-15 protein. As a result, the proportion of T_SCM_-like CAR-T cells is increased, thereby enhancing their capability to attack CAFs within the TME of solid tumors.

Despite extensive research on TME-targeting CAR-T therapies, limitations persist regarding effector cell longevity within solid tumors, and the combined application of IL-15 and CAR-T cells holds considerable potential for further development. In this study, we focused on CAFs, the predominant stromal component of the TME, as there is an urgent need for CAF-directed immunotherapy. To address the poor efficacy of CAR-T therapy in solid tumors, we aimed to engineer CAR-T cells capable of precisely disrupting the TME while enhancing survival, proliferation, and differentiation functions. Our strategy was to generate CAR-T cells (FAP/IL-15 CAR-T cells) that target FAP and autonomously synthesize and secrete IL-15 protein, thereby enhancing their capability to attack CAFs within the TME of solid tumors (Fig. 1). This dual-action design enhances anti-CAF functions within the TME—an approach unreported in prior studies. We hypothesized that FAP/IL-15 CAR-T cells would specifically recognize and eliminate both FAP-positive tumor cells and CAFs, thereby disrupting stromal components essential for solid tumor progression. Critically, the CAR construct incorporates an IL-15 sequence, enabling IL-15 secretion upon CAR gene expression via a P2A self-cleaving peptide. This feature promotes proliferation, activation, functional enhancement, and memory subset formation of CAR-T cells for solid tumor treatment. Compared to CAR-T therapy combined with exogenous IL-15 administration, this endogenous secretion strategy offers distinct advantages in maintaining effective drug concentrations in blood and tumor sites, relieving TME-mediated immunosuppression, extending intratumoral effector cell survival, and enabling long-term therapeutic responses to CAR-T cell therapy.

## Materials and methods

### Animals and cells

Female BALB/c and NOD/SCID mice (6–8 weeks old) used in this research were obtained from Guangzhou Fushuge Biotechnology Co., Ltd. (Guangzhou, China), and were maintained under pathogen-free laboratory conditions. All experimental animal procedures adhered to guidelines approved by the Animal Ethics Committee at Hainan Medical University. Animal experiments complied with ARRIVE guidelines and followed the National Institutes of Health Guide for the Care and Use of Laboratory Animals. The human glioblastoma cell line (U87) and human hepatoblastoma cell line (HepG2) were purchased from the American Type Culture Collection (ATCC). To generate FAP-expressing cells, FAP-negative HepG2 cells were transduced with lentivirus encoding human FAP cDNA and designated HepG2-hFAP. Cells were cultured in Dulbecco’s Modified Eagle Medium (DMEM; Invitrogen, USA) supplemented with 10% fetal bovine serum (FBS; HyClone, USA) and 1% penicillin/streptomycin (Invitrogen, USA). Procedures used to generate and culture CD8^+^ T cells were based on previously described methods [25, 26]. Written informed consent was obtained from all volunteers. This study was approved by the local ethics committee of Hainan Medical University.

### Isolation and identification of CAFs

Three individuals diagnosed with hepatocellular carcinoma and scheduled to undergo surgical removal at the First Affiliated Hospital of Hainan Medical University from October 2020 to January 2024 participated in the present research. All participants provided informed consent prior to enrollment, and ethical approval was secured from the institutional ethics review board at Hainan Medical University. Following resection, tumor samples were sterilized briefly with 75% ethanol, rinsed twice in sterile PBS, and finely fragmented into small tissue pieces. Subsequently, tissue fragments were transferred into centrifuge tubes containing collagenase digestion solution (DMEM medium supplemented with 0.2% collagenase A, 0.2% trypsin, 5% FBS, and gentamicin at 5 μg/mL) and incubated at ambient temperature without agitation for five minutes. The supernatant enriched with stromal cells was carefully aspirated and centrifuged at 250 × g for five minutes. The resulting cell pellet was gently resuspended in DMEM with 10% FBS and seeded into culture dishes for initial cultivation. Primary fibroblasts were isolated and propagated by employing identical protocols involving enzymatic digestion, selective sedimentation, plating, and culture in conditions optimized to promote fibroblast proliferation. Fibroblasts obtained were subsequently expanded into two 15-cm Petri dishes, cultured continuously for approximately 8–10 days, and passaged after achieving 2–3 population doublings. CAF-specific markers were assessed by flow cytometry, immunofluorescence staining (FAP), and western blot analysis (FAP, α-SMA, fibronectin.

### Western blotting (WB)

The expression of FAP, α-SMA, and fibronectin in CAFs was detected by WB using horseradish peroxidase (HRP)-conjugated antibodies (Abcam ab207178, ABclonal A17910, Proteintech 66042-1-lg). Total protein was extracted from lysed CAFs, and concentrations were determined. For immunoblotting, extracted protein samples were transferred onto PVDF membranes using wet transfer equipment (Bio-Rad, USA). Membranes were blocked in skim milk solution (5%) and incubated overnight at a 1:3000 dilution with corresponding HRP-labeled antibodies. Protein bands were subsequently visualized using BeyoECL Plus chemiluminescence reagent (Beyotime, China), and densitometric quantification was performed utilizing Bio-Rad Image Lab software.

### Production of CAR vectors and lentivirus packaging

The CAR construct included the FAP receptor, CD28 transmembrane domain, and co-stimulatory domains derived from 4-1BB and CD3 Zeta chains, packaged into a lentiviral vector. The cloning of each CAR construct was verified by sequencing. Lentiviruses were packaged using a three-plasmid expression system consisting of the recombinant plasmid and two helper plasmids (Helper 1.0 and Helper 2.0). After lentivirus packaging, CD8^+^ T cells were transduced with CAR lentiviruses. The transduction efficiency was evaluated by flow cytometry and fluorescence microscopy.

### Flow cytometry

CAR-T cells and target cells (HepG2-hFAP, HepG2, U87, or CAF cells pre-treated with mitomycin) were co-incubated at a ratio of 1:1 for 48 hours. Following incubation and washing steps, cells were labeled with phycoerythrin (PE)-conjugated anti-CD69, anti-CD25, and anti-CD62L antibodies (Sungene Biotech, China) and then subjected to flow cytometric analysis using a Beckman Coulter flow cytometer (Beckman Coulter, Inc., Brea, CA, USA).

### ELISA and ELISPOT

CAR-T cells were cultured with their respective target cells (HepG2-hFAP, HepG2, U87, or CAFs) for 16 hours, after which cell supernatants were harvested. Concentrations of interleukin (IL)-2, IL-10, MIP-1α, and tumor necrosis factor-alpha (TNF-α) were quantified using commercially available human ELISA kits (Dakewe Biosciences) following the vendor’s guidelines. Additionally, ELISPOT assays were conducted to measure interferon-gamma (IFN-γ) secretion. Briefly, control effector or FAP/IL-15 CAR-T cells (3 × 10^5^ cells/well) and irradiated target cells (1 × 10^5^ cells/well) were seeded into 96-well ELISPOT plates in triplicate and cultured at 37°C overnight. Captured IFN-γ was subsequently incubated with biotinylated anti-IFN-γ antibodies at 4°C overnight. IFN-γ-positive immunocomplexes were detected using streptavidin-AP, followed by substrate addition (BCIP/NBT). Spots generated in each well were counted using the CTL ImmunoSpot S6 Ultimate-V Analyzer.

### Proliferation and cytotoxicity assays

CAR-T cells from all experimental conditions were stained with PKH26 dye (Sigma, USA) for five minutes. After removing unbound dye, labeled cells were resuspended in complete DMEM medium and incubated at varying effector-to-target ratios (1:1, 4:1, 8:1) for 120 hours alongside HepG2-hFAP, HepG2, U87, or CAF cells (previously irradiated at 100 Gy). Flow cytometry was performed subsequently to assess CAR-T cell proliferation. Cytotoxicity assessments involved labeling target cells sequentially with PKH26 and 7-aminoactinomycin D (7-AAD), followed by incubation with CAR-T cells for six hours. Flow cytometric analysis determined the percentage of target cells eliminated by CAR-T cells.

For intracellular IFN-γ and CD107a analysis, CAR-T cells were incubated with CAFs at a 20:1 effector-to-target ratio for six hours. Subsequently, the cells were labeled using PE-conjugated anti-CD107a antibodies (BD Biosciences, USA). Intracellular staining for IFN-γ was performed after treatment with monensin (Beyotime, China), followed by fixation and permeabilization using Cytofix/Cytoperm™ kits (BD Biosciences, USA), and staining with anti-human IFN-γ monoclonal antibodies. Percentages of positively stained cells were measured by flow cytometry.

### Patient-derived tumor xenograft (PDX) modeling and in vivo treatment

To evaluate the therapeutic effects of FAP/IL-15 CAR-T cells against solid tumors in vivo, we constructed a PDX mouse model of primary hepatic carcinoma. Three patients diagnosed with hepatocellular carcinoma provided tumor tissues from surgical resections after obtaining informed consent.

The study was approved by the Institutional Ethics Committee of Hainan Medical University. Collected tumor tissues were sectioned into 3–4 mm pieces and implanted subcutaneously into the left armpits of anesthetized six-week-old male NOD/SCID mice. After tumors grew to a sufficient size, mice were euthanized, and tumors were excised, dissected under sterile conditions, and transplanted subcutaneously into additional NOD/SCID mice to continue passaging. When tumor size reached around 100 mm^3^, mice were randomly assigned to treatment groups and intravenously infused through the tail vein with CAR-T cells at a dose of 5 × 10^6^ cells per mouse. Tumor growth was assessed at three-day intervals. Upon study completion, mice were euthanized, and tumors were collected for further investigation. For humane euthanasia, animals were transferred to a carbon dioxide chamber, initiating gas delivery at 40% chamber volume displacement per minute, gradually rising to 100%, until mice became unconscious and subsequently expired. Individual confirmation of death was ensured via cervical dislocation immediately thereafter.

### In vivo treatment monitored by imaging

To further confirm tumor suppression in mice following FAP/IL-15 CAR-T cell treatment, we established xenograft models using the HepG2-luc cell line. To establish tumor xenografts, NOD/SCID mice were injected intravenously with HepG2-luc cells (1 × 10⁶ per mouse). Following CAR-T therapy administration, tumor progression was monitored through bioluminescence imaging (BLI) conducted every third day for a duration of 27 days. During imaging sessions, mice received intraperitoneal injections of D-luciferin potassium solution (200 μL at a concentration of 15.15 mg/mL), and photon emission intensities (photons/s/cm^2^/sr) at tumor sites were quantitatively analyzed using Living Image 3.2 software. Tumors were collected from mice after the final imaging session for further ex vivo photography.

### Immunohistochemistry (IHC)

Tumor tissue sections were subjected to immunostaining procedures to evaluate tumor cell proliferation, CAR-T cell infiltration, and vascular structures, using antibodies targeting Ki-67 (Maixin Biotech, China), FAP (R&D, USA), collagen I (Abcam, USA), and CD31 (Abcam, USA). Apoptosis within tumor tissues was assessed by a TUNEL immunofluorescence assay (FITC, in situ Cell Death Detection Kit; Roche, Switzerland), performed according to the manufacturer’s recommended protocol. Fluorescent microscopy images were acquired with a Nikon microscope (Nikon, Japan).

### In vivo survival and antitumor efficacy of CAR-T cells

On days 7, 14, and 28 after treatment, three primary tumor-bearing mice from each group were euthanized. Peripheral blood, spleens, and tumor tissues were collected. The tissues were minced, homogenized, and analyzed by flow cytometry to determine CD3 expression ratios. On day 28, serum levels of TNF-α and IFN-γ were measured by ELISA.

### RNA-seq assay

RNA sequencing (RNA-seq) was performed by Shanghai Meiji Biomedical Technology Co., Ltd. (Shanghai, China). Total RNA was extracted from tumor tissues of PDX mouse models using TRIzol reagent (Invitrogen, USA) following the manufacturer’s protocol. RNA integrity was evaluated by 1.5% agarose gel electrophoresis, and samples displaying a 28S:18S rRNA ratio of approximately 2:1 were considered sufficiently intact. RNA purity was assessed using a Nanodrop microspectrophotometer, with samples having A260/A280 ratios of 1.8 to 2.0 deemed acceptable. Eukaryotic mRNA sequencing was performed on a NovaSeq X Plus platform using the Illumina NovaSeq Reagent Kit. Library construction involved poly-A RNA capture, RNA fragmentation, first- and second-strand cDNA synthesis, adaptor ligation, and sequencing. Reads mapping was normalized to transcripts per million (TPM). Differentially expressed genes (DEGs) were identified by a fold change of >2 and an adjusted *p*-value < 0.05.

### Toxicity assessment

BALB/c mice were allocated into three experimental groups receiving intravenous injections of either FAP/IL-15 CAR-T cells, FAP CAR-T cells, or PBS vehicle as controls. At the conclusion of the experimental period, mice underwent euthanasia, and essential organs, including heart, liver, kidney, lungs, spleen, and brain, were excised. Tissues were embedded in paraffin, sectioned to 4 μm thickness, stained with hematoxylin-eosin (H&E), and histologically analyzed using a Nikon microscope (Nikon, Japan). Blood samples were obtained for biochemical analysis utilizing an AU5800 Clinical Chemistry Analyzer (Beckman Coulter, Inc.), focusing on the evaluation of liver and cardiac biomarkers, such as alanine aminotransferase (ALT), aspartate aminotransferase (AST), creatine kinase (CK), CK-MB, and lactate dehydrogenase (LDH-L).

### Statistical analysis

Experimental data were initially recorded in Excel spreadsheets and subsequently analyzed utilizing GraphPad Prism version 9.0 (GraphPad Software, La Jolla, CA, USA). Statistical outcomes are displayed as mean ± standard deviation (SD). Multiple-group comparisons were performed by one-way analysis of variance (ANOVA), whereas two-group differences were evaluated by Student’s t-tests. Kaplan-Meier analyses were employed to represent survival differences among treatment groups. Differences were considered statistically meaningful at *p*-values lower than 0.05.

## Results

### Generation and characterization of FAP/IL-15 CAR-T cells

**Fig. 2:**
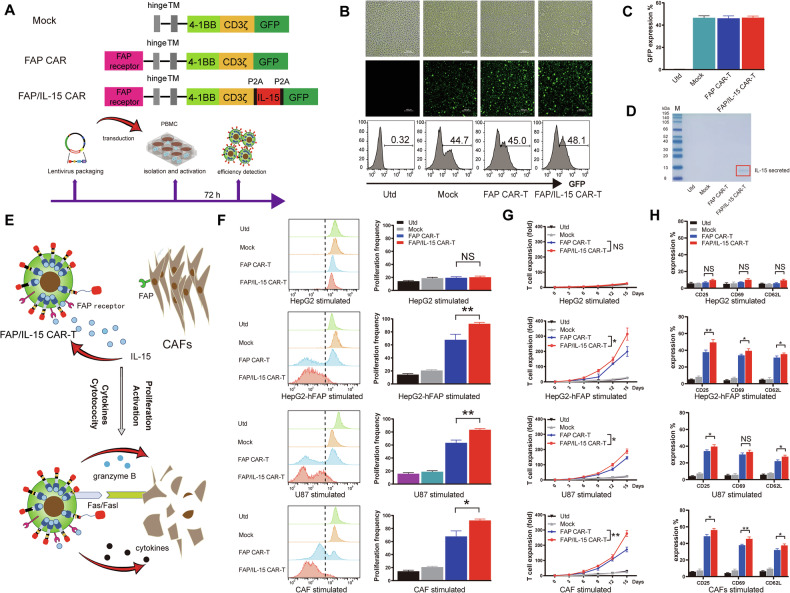
Generation and characterization of FAP/IL-15 CAR-T cells. **A** The schematic of the CAR construct in each group. **B** The GFP expression of CAR-T cells was detected using fluorescence microscopy and flow cytometry after being transduced with either the Mock, FAP CAR or FAP/IL-15 CAR. Magnification, 400×. **C** Quantitative analysis of GFP expression in CAR T cells using flow cytometry. *n* = 3. **D** the content of IL-15 in the supernatant of CAR-T cells from each group were determined using SDS-PAGE. **E** The schematic of FAP/IL-15 CAR-T cells specifically eliminate CAFs upon stimulation with FAP antigens. **F** Flow cytometry analysis of proliferation frequency in PKH26-labeled CAR T cells co-cultured with irradiated HepG2-FAP, HepG2, U87 or CAFs. *n* = 3, *NS* represents not significant, *P < 0.05, **P < 0.01. **G** Quantitative analysis of proliferation frequency of CAR T cells in each group. *n* = 3, NS represents not significant, *P < 0.05, **P < 0.01. **H** The CAR T cells in each group were co-culture with HepG2, HepG2-FAP, U87 or CAFs for 48 h, then stained with PE-conjugated anti-CD62L, anti-CD69 or anti-CD25 mAb and analyzed by flow cytometry for the activation. *n* = 3, *P < 0.05, **P < 0.01.

A pLent-EF1a-FH-CMV-copGFP-P2A-Puro vector (Supplementary Fig. 1A) was digested with restriction enzymes and analyzed using agarose gel electrophoresis (Supplementary Fig. 1B). The digested vector was recombined with Mock, FAP CAR, or FAP/IL-15 CAR genes (Fig. 2A) and subsequently transformed into E. coli. Individual colonies were selected, and insertion of the CAR gene was confirmed by PCR. Sequencing verified that the cloned CAR fragments matched the intended design. The constructed CAR vector plasmid was transfected into 293 T cells and cultured until GFP fluorescence was detected (Supplementary Fig. 1C). The supernatant was concentrated and purified to obtain high-quality lentivirus (2 × 10^8^ TU/mL).

Sorted CD8^+^ T cells were activated and transduced with lentivirus to generate CAR-T cells. Significant GFP fluorescence was observed by fluorescence microscopy on day three post-transduction. Flow cytometry was utilized to measure transduction efficiency by detecting GFP expression (Fig. 2B). Both CAR-T cell groups exhibited transduction efficiencies above 45% (Fig. 2C). To determine IL-15 secretion by FAP/IL-15 CAR-T cells, SDS-PAGE was performed and confirmed the presence of IL-15 (His-tagged) in the supernatant (Fig. 2D and Supplementary Fig. 4).

### FAP/IL-15 CAR-T cells showed FAP antigen-dependent activation and proliferation in vitro

CAR-expressing T cells recognize specific antigens independently of major histocompatibility complex (MHC) presentation, thereby circumventing conventional activation requirements of physiological T cells and promoting CAR-T cell activation and proliferation (Fig. 2E).

To evaluate CAR-T cell proliferation following FAP antigen stimulation, effector cells were co-cultured with irradiated HepG2-hFAP, HepG2, U87 cells, or CAFs. Proliferation of FAP/IL-15 CAR-T cells was significantly greater compared to controls, including FAP CAR-T cells (Fig. 2F, G). Flow cytometry analysis indicated that activation markers (CD25, CD69, CD62L) were significantly upregulated in FAP/IL-15 CAR-T cells compared to negative controls and even FAP CAR-T cells (Fig. 2H). Thus, CAR-T cells targeting FAP and secreting IL-15 demonstrated robust proliferation and activation in response to stimulation with FAP-positive target cells.

### FAP/IL-15 CAR-T cells recognize and eliminate FAP-positive targets by secreting inflammatory cytokines

**Fig. 3:**
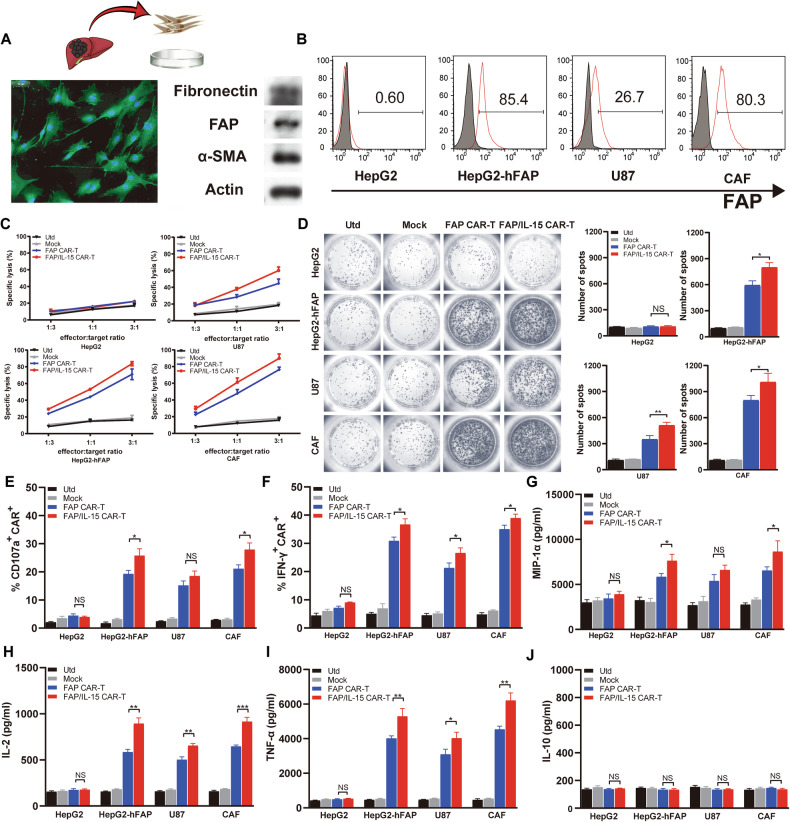
FAP/IL-15 CAR-T cells specifically eliminate FAP positive target cells by inflammatory cytokine secretion. **A** After seperating and purifying CAFs from primary human liver cancer tissues, their specific markers were identified by using Western Blot(FAP, α-SMA, Fibronectin) and immunofluorescence assays(FAP). **B** The FAP expression of each target cells was detected by flow cytometr. **C** The CAR cells were co-incubated with PKH26-prestained CAFs, HepG2-hFAP, HepG2 or U87 cells at E/T ratio 1:3, 1:1 or 3:1, for 6 hours, PI was used for lysed cell staining. The ratios of PHK26^+^ PI^+^ cell were measured by flow cytometry. *n* = 3. **D** The secretion of IFN-γ in the CAR T cells of different groups against the stimulations of target cells was detected by ELISPOT assay to indicate the increased amount of IFN-γ-producing CAR cells. Representative image of ELISPOT plate readout analyzing the frequency of IFN-γ-secreted CAR T cells are shown histograms represent data of the triplicates for 3 × 10^5^ cells from three independent ELISPOT assays, and shown as bars of means + S.D. *n* = 3, NS represents not significant, *P < 0.05, ** *P* < 0.01. **E**, **F** After co-culturing with CAFs at a 20:1 ratio for 6 h, the IFN-γ(intracellular) and CD107a expression of CAR cells in each group were measured by flow cytometry. *n* = 3, *P < 0.05. **G**–**J** After co-culture with CAR T cells and CAFs, supernatants in each group were separated and analyzed for secretion of TNF-a, IL-2, MIP-1α and IL-10 and measuring by the ELISA kits. Results indicated the increased production of above three cytokines from the CAR T cells Stimulated by target cells. Bar graphs showmean ofcytokine concentration + S.D. *n* = 3, NS represents not significa*n*t, *P < 0.05, **P < 0.01, ***P < 0.001.

In cytotoxicity assays, human CAFs isolated from tumor tissues served as target cells. CAF identification was performed by WB (FAP, α-SMA, Fibronectin), immunofluorescence, and flow cytometry (FAP expression) (Fig. 3A, B and Supplementary Fig. 5). Cytotoxicity assays demonstrated that FAP-targeting CAR-T cells specifically eliminated FAP-positive cells, including CAFs, in an effector-target ratio-dependent manner. Notably, cytotoxic activity was markedly stronger in FAP/IL-15 CAR-T cells relative to control groups and FAP CAR-T cells (Fig. 3C). To validate this observation and further investigate their killing capacity against FAP-expressing targets, we performed flow cytometric analysis of intracellular IFN-γ levels and assessed CD107a translocation, an indicator of cytotoxic cell activity. Results indicated significantly upregulated IFN-γ and CD107a expression in FAP/IL-15 CAR-T cells, consistent with cytotoxicity assay outcomes (Fig. 3E, F). Additionally, ELISPOT assays revealed elevated numbers of IFN-γ-secreting spots in the FAP/IL-15 CAR-T group compared with Utd, Mock, and FAP CAR-T groups after stimulation with corresponding target cells (Fig. 3D).

Next, to characterize cytokine production following CAR-T cell activation, culture supernatants from activated FAP/IL-15 CAR-T cells were subjected to ELISA. Upon co-incubation with FAP-positive target cells, FAP/IL-15 CAR-T cells secreted significantly elevated levels of inflammatory cytokines such as TNF-α, IL-2, and MIP-1α relative to the controls (Fig. 3G–I). Conversely, IL-10 concentrations remained comparable across all experimental groups (Fig. 3J, *p* > 0.05). Collectively, these data suggest that stimulation by FAP antigen substantially increases pro-inflammatory cytokine secretion by FAP/IL-15 CAR-T cells, thus facilitating localized inflammatory responses and efficient tumor targeting.

### FAP/IL-15 CAR-T cells exhibit enhanced antitumor effects in xenograft models

**Fig. 4:**
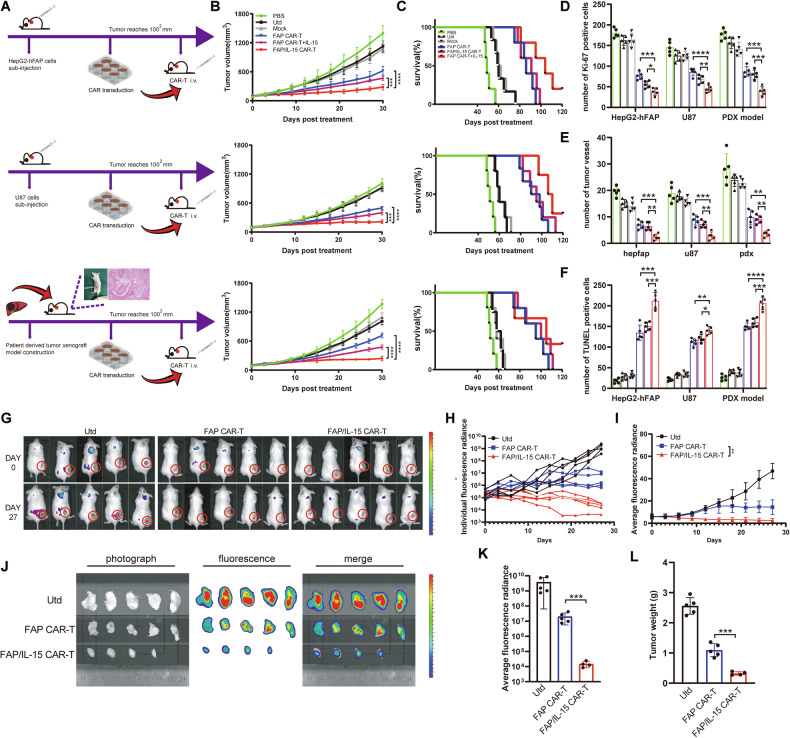
FAP/IL-15 CAR-T cells treatment lead to a superior tumor inhibition effect in xenograft mice. **A** The schematic of subcutaneous tumor-bearing mouse model construction and CAR-T therapy. **B** The tumor size of tumor-bearing mice was measured. *n* = 5, *** *P* < 0.001, **** *P* < 0.0001. **C** Kaplan-Meier survival curve of each group. *n* = 5. **D** Ki-67 expression of tumor tissue of each group were detection by immunohistochemical stain. *n* = 5, *P < 0.05, **P < 0.01, ***P < 0.001, ****P < 0.0001. **E** The number of tumor vessels perfield were detection by immunohistochemical stain. *n* = 5, **P < 0.01, ***P < 0.001. **F** The number of apoptosis cells perfield were detection by TUNEL. *n* = *5*, *P < 0.05, **P < 0.01, ***P < 0.001, ****P < 0.0001. **G** Quantitative bioluminescence (radia*n*ce=photons/cm^2^/sr) imaging data for HepG2-luc subcutaneous transplanted mice after receiving CAR-T treatment. **H**, **I** Tumor growth is displayed for individual mice (**H**) and as average values (**I**). *n* = *5*, **P < 0.01. **J** Quantitative bioluminescence (radiance=photons/cm^2^/sr) imaging data for the tumor that was removed after the monitoring process was finished. **K** Quantitative analysis of tumor bioluminescence values. *n* = *5*, ***P < 0.001. **L** Quantitative analysis of tumor weight. *n* = *5*, ***P < 0.001.

To further investigate the mechanism underlying tumor growth inhibition by FAP/IL-15 CAR-T cell therapy, xenograft models using HepG2-hFAP, U87, and primary hepatic carcinoma cells were established (Fig. 4A). Our data indicated that FAP/IL-15 CAR-T cell treatment significantly suppressed tumor growth and prolonged survival in xenograft-bearing mice compared to FAP CAR-T cells, FAP CAR-T cells combined with exogenous IL-15, and other controls (Fig. 4B, C). In vivo imaging demonstrated distinct luciferase fluorescence at tumor sites 15 minutes after intravenous administration of D-luciferin potassium. Compared with the rapid tumor progression observed in untreated (Utd) mice, tumors in FAP/IL-15 CAR-T-treated mice exhibited minimal or no growth, suggesting enhanced antitumor activity via precise targeting of FAP-positive cells and IL-15 secretion by effector cells (Fig. 4G–L).

To confirm the antitumor mechanism of FAP/IL-15 CAR-T cells, tumor tissues from xenograft models were analyzed immunohistochemically for Ki-67, vessel density (CD31), and apoptosis (TUNEL assay). Additionally, immunostaining results showed significantly reduced expression of proliferation marker Ki-67 and endothelial marker CD31 within the FAP/IL-15 CAR-T group in comparison to PBS, untreated, FAP CAR-T, and FAP CAR-T combined with exogenous IL-15 treatment groups (Fig. 4D, E). This suppression of proliferation and angiogenesis correlated with enhanced apoptotic activity as evidenced by the TUNEL assay (Fig. 4F). Collectively, these findings support the conclusion that adoptive transfer of FAP/IL-15 CAR-T cells effectively inhibits tumor growth by concurrently promoting apoptosis and restraining both angiogenic activity and tumor cell proliferation. Furthermore, to clarify whether targeted CAF elimination was responsible for tumor inhibition by FAP/IL-15 CAR-T cells, we analyzed the expression of FAP and collagen type I in treated tumor tissues. Results confirmed this proposed mechanism (Supplementary Fig. 2A–B). Lastly, we evaluated potential therapy-associated toxicity four weeks post-final treatment. Histological analyses using hematoxylin-eosin staining showed no apparent tissue necrosis or inflammatory infiltration in any examined samples (Supplementary Fig. 3A). Consistent with this observation, serum biochemical evaluations revealed no statistically significant variations between mice administered FAP/IL-15 CAR-T cells and PBS controls (Supplementary Fig. 3B). These results collectively suggest that FAP/IL-15 CAR-T cell therapy exhibits minimal systemic toxicity.

### Long-term antigen-dependent persistence of FAP/IL-15 CAR-T cells accompanied by a distinct phenotypic profile

**Fig. 5:**
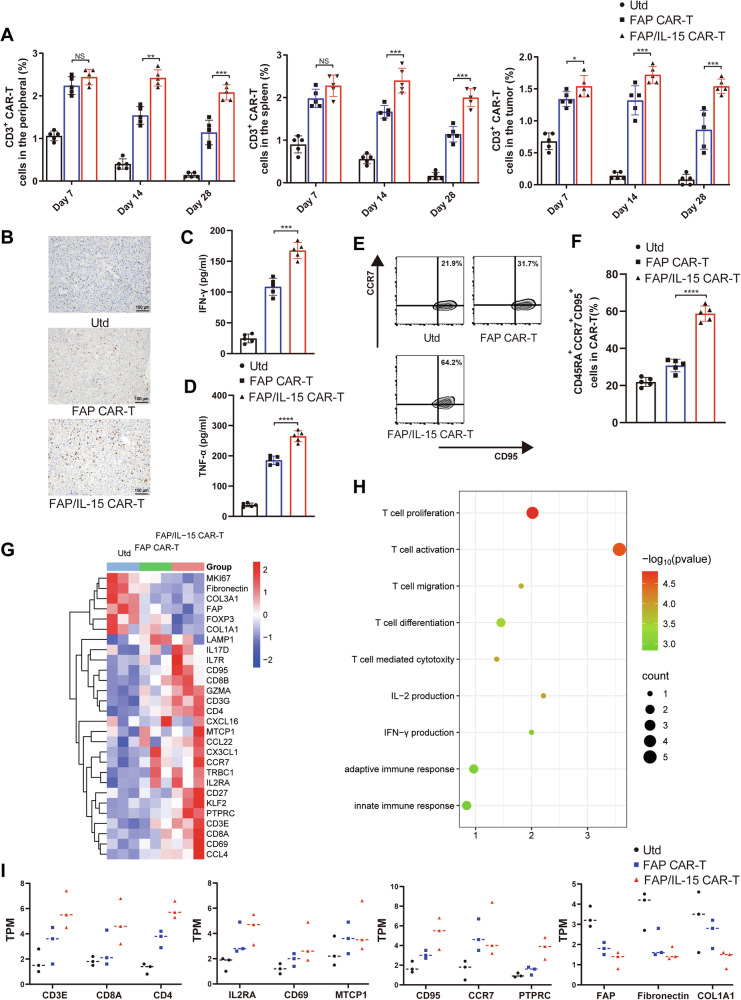
Long-term antigen-dependent persistence of FAP/IL-15 CAR-T cells in tumor-bearing mice. **A** The percentage of human CD3^+^ in tumor, spleen, and peripheral blood of PDX mice that were adoptively transferred with CAR T cells were measured by flow cytometry on days 7, 14, and 28. *n* = 3, NS represents not significant, *P < 0.05, **P < 0.01, ***P < 0.001. **B** CD3 expression of tumor tissue of each group were detection by immunohistochemical stain (200x, Scale bar=100 um). **C**, **D** Human IFN-γ and TNF-α levels in tumor-bearing mice serum were detected by ELISA after adoptively transferring with CAR-T cells in each groups. *n* = 5, ***P < 0.001, ****P < 0.0001. **E**, **F** Flow cytometry analysis shows changes in CD45RA^+^, CCR7^+^, CA95^+^ CAR-T cells in each groups, *n* = 5, ****P < 0.0001. **G** Heatmap of the candidate genes i*n* tumor tissues from PDX mice that received Utd, FAP CAR-T and FAP/IL-15 CAR-T treatment. **H** GO enrichment analysis of candidate genes in tumor tissues from PDX mice that received Utd, FAP CAR-T and FAP/IL-15 CAR-T treatment. **I** TPM was used for relative assessment of candidate gene expression levels, such as T cell activation, proliferation, T_SCM_ subsetsand and tumor-associated fibroblasts-associated genes (*n* = 3 independent biological samples).

The in vivo persistence of FAP/IL-15 CAR-T cells was evaluated on days 7, 14, and 28 post-treatment by detecting CD3 expression in spleen and tumor tissues using flow cytometry. CD3^+^ CAR-T cells persisted throughout the experimental period in the FAP/IL-15 CAR-T group. Although CD3^+^ CAR-T cell numbers decreased slightly in tumors, blood, and spleen by day 28, they retained robust antitumor activity (Fig. 5A). This persistence was corroborated by immunohistochemical staining of CD3 in tumor tissues (Fig. 5B). Moreover, serum cytokine assays conducted on day 28 revealed substantially elevated human IFN-γ and TNF-α concentrations in animals treated with FAP/IL-15 CAR-T cells compared to other treatment groups (Fig. 5C, D). These findings suggest that the engineered FAP/IL-15 CAR-T cells persist robustly in vivo, continuously secreting pro-inflammatory cytokines that contribute to sustained suppression of tumor growth.

To further characterize the phenotype of FAP/IL-15 CAR-T cells in vivo, we assessed phenotypic changes in these cells targeting CAFs and secreting IL-15. Our data revealed that FAP/IL-15 CAR-T cells exhibited an IL-15-driven phenotype, characterized by an increased proportion of T_SCM_ cells (CCR7^+^CD45RA^+^CD95^+^), enhancing their proliferative potential upon antigen stimulation (Fig. 5E, F). RNA-seq analyses of tumor tissues from PDX models (Utd, FAP CAR-T, and FAP/IL-15 CAR-T groups) were conducted to profile gene expression changes. Gene Ontology (GO) enrichment analysis of differentially expressed genes (DEGs) showed significant enrichment of genes involved in T-cell proliferation, activation, mediated cytotoxicity, and IFN-γ and IL-2 production in the FAP/IL-15 CAR-T group (Fig. 5G, H). Gene signature analysis confirmed upregulation of genes associated with CAR-T cell activation, proliferation, cytotoxicity, and the T_SCM_ phenotype in the FAP/IL-15 CAR-T group compared to controls. Moreover, tumor-associated (MKI67) and CAF-related (FAP, Fibronectin, COL1A1) genes were downregulated in the FAP/IL-15 CAR-T group compared to other groups (Fig. 5I). These data collectively indicate that FAP-targeting CAR-T cells demonstrate enhanced activation and cytotoxic functions upon antigen stimulation. Additionally, endogenous IL-15 secretion significantly enhances effector cell viability, enabling effective tumor suppression in mice through targeted elimination of CAFs in solid tumors.

## Discussion

Recently, IL-15 has attracted considerable attention in cancer immunotherapy due to its significant immunomodulatory effects. As part of the IL-2 cytokine family, IL-15 exhibits overlapping functional roles with IL-2 in modulating both adaptive and innate immune responses [27]. IL-15 initiates signaling by forming a complex upon interaction with its unique receptor subunit, IL-15Rα, a transmembrane receptor protein. Subsequently, this complex associates with the IL-2Rβ/γ heterodimer, which is expressed on multiple immune effector cells such as B cells, natural killer (NK) cells, and effector T cells. Activation of this signaling pathway enhances the maturation, viability, and antitumor cytotoxicity of these immune populations [27–29]. Nonetheless, a significant barrier arises due to the limited cellular sources of IL-15. Typically, IL-15 secretion following immune stimulation occurs predominantly from stromal cells, endothelial cells, dendritic cells (DCs), monocytes, macrophages, and renal epithelial cells, but rarely originates from NK or T cells themselves [30, 31]. As a result, cancer patients exhibiting weakened immune systems who receive adoptively transferred NK or T cell therapies frequently lack sufficient intrinsic IL-15 support, restricting the persistence and activation of therapeutic cells [32]. Indeed, adequate intratumoral IL-15 levels within the TME are essential for optimal antitumor efficacy, with diminished local IL-15 production closely linked to increased tumor relapse rates and decreased patient survival outcomes [33]. Exogenous IL-15 administration as an activator or adjuvant in adoptive immune-cell therapy is increasingly recognized as theoretically sound, with several such strategies now progressing through clinical trials [34].

However, the clinical utility of IL-15 remains limited by its short half-life, primarily due to rapid renal clearance. Simply increasing the dosage does not resolve this issue and complicates balancing efficacy and safety in combination therapies [35]. To enhance bioavailability, several strategies have been explored, such as domain modifications or fusion proteins including IL-15/IL-15Rα complexes and PEGylated IL-15 (IL-15-PEG), aimed at prolonging IL-15’s half-life [36, 37]. Nevertheless, these approaches remain hindered by the tumor-stromal barrier within the TME of solid tumors. Dysregulation of the ECM, elevated interstitial fluid pressure, and abnormal neovascularization within tumor stroma collectively restrict uniform drug diffusion throughout the tumor region [38]. Consequently, infiltrating CAR-T cells encounter insufficient IL-15 stimulation within tumors. Previous studies on multiple IL-15-based CAR-T therapies targeting GPC-3 [39] or CLDN18.2 [40] have primarily focused on directly attacking tumor cells. However, this approach has not effectively addressed the challenge of achieving sufficient IL-15 infiltration within the tumor microenvironment. To address this limitation, we designed CAR-T cells capable of constitutively encoding and secreting IL-15 protein, aiming to enhance local IL-15 concentrations and improve CAR-T activation within the TME. As anticipated, our results demonstrated that FAP/IL-15 CAR-T cells exhibited enhanced proliferation compared to controls in vitro due to IL-15 secretion. Additionally, these CAR-T cells achieved greater intratumoral IL-15 concentrations in the PDX tumor model compared to the combination of CAR-T cells and exogenous IL-15 administration, confirming the therapeutic advantage of endogenous IL-15 secretion in preclinical models. This suggests that IL-15 secretion by FAP/IL-15 CAR-T cells is efficient, stable, and capable of overcoming uneven drug distribution caused by the tumor-stromal barrier. CAFs, the predominant stromal cell population within solid tumors, exhibit contractile properties, thereby compressing tumor tissue and significantly contributing to tumor-stromal barrier formation [41]. This contraction is also a primary factor elevating interstitial pressure within tumor stroma [42]. Additionally, CAFs secrete ECM remodeling enzymes and deposit substantial amounts of collagen (mainly types I, IV, XI, and XII) and fibronectin, reshaping the ECM and forming a physical barrier that shields tumor cells. This barrier indirectly contributes to resistance against chemotherapy, radiotherapy, and immunotherapy [43]. Given their critical role in shaping the TME, CAF-targeted immunotherapy has become a promising therapeutic strategy for solid tumors, necessitating the rapid development of effective therapies. Although prior studies have described FAP-specific CAR-T cell therapies [44], we reconstructed an analogous CAR-T platform to serve as an internal control in the current study. Our results indicated that engineered FAP/IL-15 CAR-T cells exhibited notably improved cellular activation, proliferation, cytokine release, and cytotoxic potency compared to controls upon exposure to FAP-expressing target cells in vitro. Additionally, these engineered CAR-T cells consistently displayed elevated proportions of CD62L^+^ subset, indicative of a central memory T-cell subset. This observation suggests that autocrine IL-15 production promotes the differentiation of FAP-specific CAR-T cells toward phenotypes characterized by heightened activation status and memory function, thus bolstering their effectiveness in eliminating CAFs and tumor cells expressing FAP. Furthermore, using in vivo tumor models established by xenografting HepG2-hFAP, U87, and primary hepatic carcinoma cell lines into mice, we validated the antitumor efficacy of FAP/IL-15 CAR-T therapy. Our data revealed significantly suppressed tumor progression and improved overall survival in mice administered FAP/IL-15 CAR-T cells relative to all other experimental cohorts. Notably, combining exogenous IL-15 with conventional FAP CAR-T cells resulted in inferior therapeutic outcomes compared to endogenous IL-15-producing CAR-T cells, likely reflecting poor bioavailability and challenges in controlling systemic IL-15 dosing. Pathological analyses confirmed significantly reduced proliferation and increased apoptosis in tumors treated with FAP/IL-15 CAR-T cells compared to the FAP CAR-T cell group. Previous studies reported that CAFs promote angiogenesis within the TME by activating the TGF-β-SMAD3 pathway and secreting VEGF along with other angiogenic cytokines [45]. CAFs are primarily located near tumor vessels, forming a protective barrier restricting the penetration of antitumor drugs [46]. Although the anti-angiogenic effects of FAP-targeted CAR-T cells remain incompletely understood, our results demonstrate that targeted elimination of CAFs by FAP CAR-T and particularly FAP/IL-15 CAR-T cells inhibited tumor angiogenesis, as evidenced by reduced CD31 expression, with FAP/IL-15 CAR-T cells showing stronger effects. In conclusion, our data demonstrate that FAP-targeted CAR-T cells, especially those engineered to secrete IL-15, exhibit potent antigen-specific recognition, rapid activation, and robust effector differentiation. This enables the selective elimination of FAP-positive tumor cells and CAFs in vitro and in vivo, disrupting pro-tumorigenic CAF functions, including angiogenesis.

Two major challenges associated with traditional TME-targeting CAR-T therapy that must be addressed are effector-cell exhaustion and therapeutic toxicity. Successful CAR-T therapy requires effector cells capable of continuous activation, differentiation, and targeted cytotoxic activity, a process that is highly demanding [47]. Persistent antigen stimulation leads to CAR-T cell exhaustion, characterized by reduced proliferative capacity and impaired effector function, ultimately causing resistance and relapse in CAR-T therapy [48]. Additionally, the accumulation of immunosuppressive cells within the TME restricts the function and activity of infiltrating CAR-T cells. For example, regulatory T cells (Tregs) create an inhibitory environment by secreting immunosuppressive cytokines and depleting IL-2 [49]. Therefore, it is critical to develop CAR constructs and production methods aimed at counteracting CAR-T cell exhaustion to improve therapeutic efficacy. T_SCM_ cells are defined by markers including CD45RA, CD45RO, CD95, CCR7, CD62L, CD122, IL7Rα, and CD27, exhibiting distinct surface-molecule expression profiles at various differentiation stages [50]. The goal of this study was to increase the proportion of T_SCM_ cells (CD45RA^+^ CCR7^+^ CD95^+^) during CAR-T cell preparation by enhancing autonomous IL-15 secretion, thereby improving the proliferation and survival of CAR-T cells in vivo. This particular subset is strongly associated with treatment responses and prevention of relapse following adoptive CAR-T therapy. Consistent with this hypothesis, our CAR-T cell survival analysis indicated that FAP/IL-15 CAR-T cells persisted for over four weeks in the spleen, peripheral blood (coinciding with an increased T_SCM_ proportion), and tumor tissues, surpassing other groups, including FAP CAR-T cells without IL-15 secretion. The extended persistence of FAP/IL-15 CAR-T cells provided durable cytotoxic effects, as evidenced by sustained high serum levels of IFN-γ and TNF-α in treated mice. Further supporting these findings, RNA-seq analyses revealed gene-expression patterns consistent with prolonged CAR-T cell persistence, confirming that the sustained presence of infused FAP/IL-15 CAR-T cells contributes to long-term remission in solid tumors.

In terms of safety, although combining IL-15 with adoptive immunotherapy is generally regarded as relatively safe, previous reports indicate that systemic administration of excessive IL-15 can induce extensive peripheral lymphocyte expansion and acute lymphocytic pneumonitis [51]. Our safety assessments demonstrated that administering FAP/IL-15 CAR-T cells in vivo is safe, despite the sustained, high intratumoral IL-15 concentrations. Because IL-15 secretion by FAP/IL-15 CAR-T cells is gradual, continuous, and controllable, we suggest that this CAR design could effectively mitigate the toxicity associated with IL-15-combined CAR-T cell therapies. In summary, this study aimed to develop immunotherapies targeting CAFs to specifically disrupt the TME in solid tumors. To address challenges inherent in co-administering IL-15 with CAR-T cells targeting the tumor microenvironment, we developed a novel CAR construct that incorporates intrinsic IL-15 secretion capacity directly into FAP-targeted CAR-T cells. This work represents the first instance reporting therapeutic effectiveness of TME-directed CAR-T cells engineered for autonomous IL-15 production against solid malignancies. Our findings provide robust preclinical evidence supporting the targeted elimination of both CAFs and tumor cells positive for FAP by FAP/IL-15 CAR-T cells, underscoring their potential utility as adoptive cellular immunotherapies. We conclude that this optimized CAR design, through continuous, gradual, and efficient intratumoral IL-15 secretion, achieves higher local cytokine concentrations, thereby overcoming previous limitations of systemic IL-15 administration regarding effector-cell survival, antigen recognition, and toxicity. We further speculate that TME disruption by CAR-T cells may dismantle the tumor-stromal barrier, facilitating improved infiltration of IL-15 and effector cells and resulting in a synergistic therapeutic effect. Although our results showing tumor regression in multiple NOD/SCID models (including both orthotopic and subcutaneous tumors) are promising, additional studies are required before clinical translation becomes feasible. Future research directions may involve developing enhanced IL-15 variants, such as IL-15 heterodimeric agonists or IL-15/IL-15Rα complexes, for incorporation into TME-directed CAR-T cell platforms. Furthermore, combined therapeutic approaches integrating chemotherapy or immune checkpoint blockade with engineered CAR-T cells merit further exploration.

## Supplementary information

supplementary figure legend

Supplementary Fig. 4

Supplementary Fig. 5

Supplementary Fig. 1

Supplementary Fig. 2

Supplementary Fig. 3

## Data Availability

The datasets generated and/or analyzed during the current study are available from the corresponding author upon reasonable request.
