# Supplementary material for: A new strategy for CAR-T therapy in solid tumors: IL-15-autocrine signaling augments tumor stroma depletion and promotes a TSCM subset in the TME
Source: Cell Death Dis. 2025 Dec 27;17(1):39. doi: 10.1038/s41419-025-08405-2 (PMC12808773; doi:10.1038/s41419-025-08405-2)
Supplement: Supplementary file 1 — supplementary figure legend [file 41419_2025_8405_MOESM1_ESM.docx]

**Supplementary Fig. 1. FAP/IL-15 CAR Recombinant vector construction and lentivirus packaging.** (A)The structure of the lentiviral vector in this study. (B)Detection of the expression of the vector by PCR (before and after enzymatic digestion). (C)After infecting HEK293T cells with the FAP/IL-15 CAR lentivirus, a distinct GFP fluorescence was observed.

**Supplementary Fig. 2. Changes in the expression levels of CAFs-related markers in tumor tissues of PDX mice after received FAP/IL-15 CAR-T cell treatment.** (A) FAP expression of tumor tissue of each group were detection by immunohistochemical stain. *n*=5. **P*<0.05, ***P*<0.01. (B)Collagen-1 area tumor tissue of each group were detection by immunohistochemical stain (400x , Scale bar=50 um). *n*=5, ***P*<0.01.

**Supplementary Fig. 3. Toxicity assessment for FAP/IL-15 CAR-T cell treatment.** (A)Serum levels of ALT, AST, CK, CK-MB and LDH-L in the indicated treatment groups. *n*=3, NS represents not significant. (B) Representative images of hematoxylin-eosin staining in the primary organs of mice at the second day (400x , Scale bar=50 um)

**Supplementary Fig. 4.** Full and uncropped SDS-PAGE.

**Supplementary Fig. 5.** Full and uncropped western blots.
