# Supplementary figures and images for: A new strategy for CAR-T therapy in solid tumors: IL-15-autocrine signaling augments tumor stroma depletion and promotes a TSCM subset in the TME
Source: Cell Death Dis. 2025 Dec 27;17(1):39. doi: 10.1038/s41419-025-08405-2 (PMC12808773; doi:10.1038/s41419-025-08405-2)

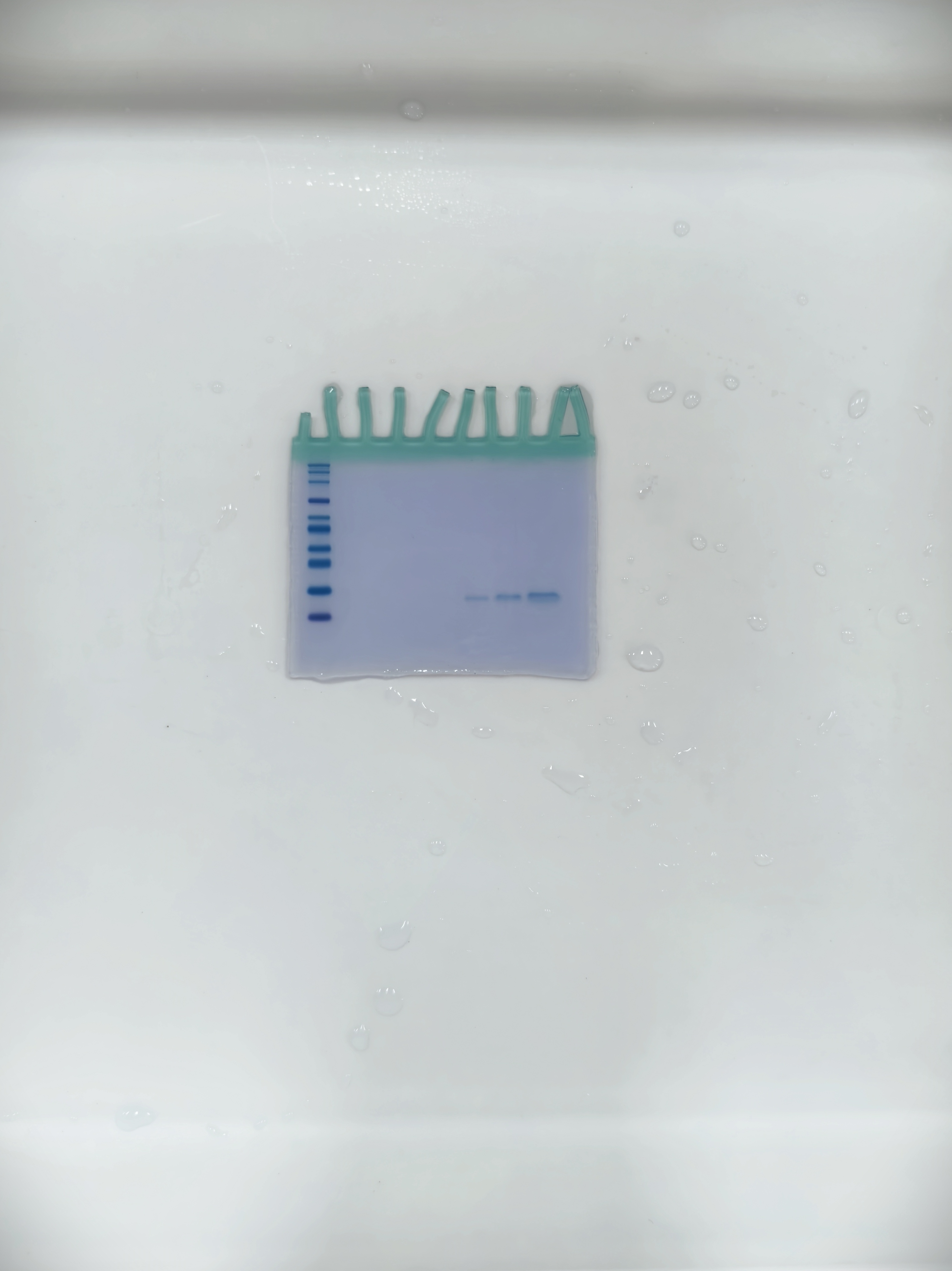

Supplement: Supplementary file 2 — Supplementary Fig. 4 [file 41419_2025_8405_MOESM2_ESM.tif]

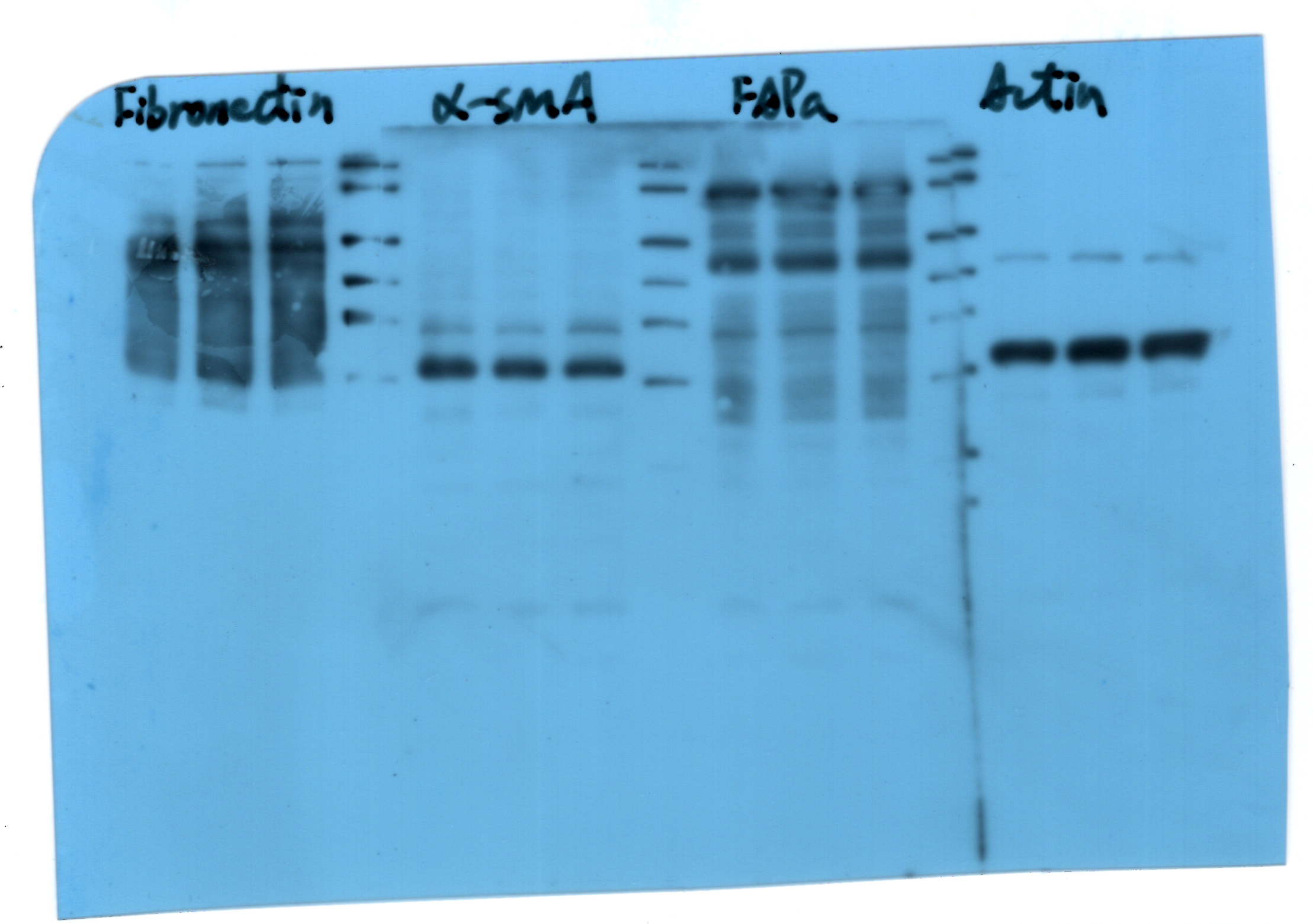

Supplement: Supplementary file 3 — Supplementary Fig. 5 [file 41419_2025_8405_MOESM3_ESM.tif]

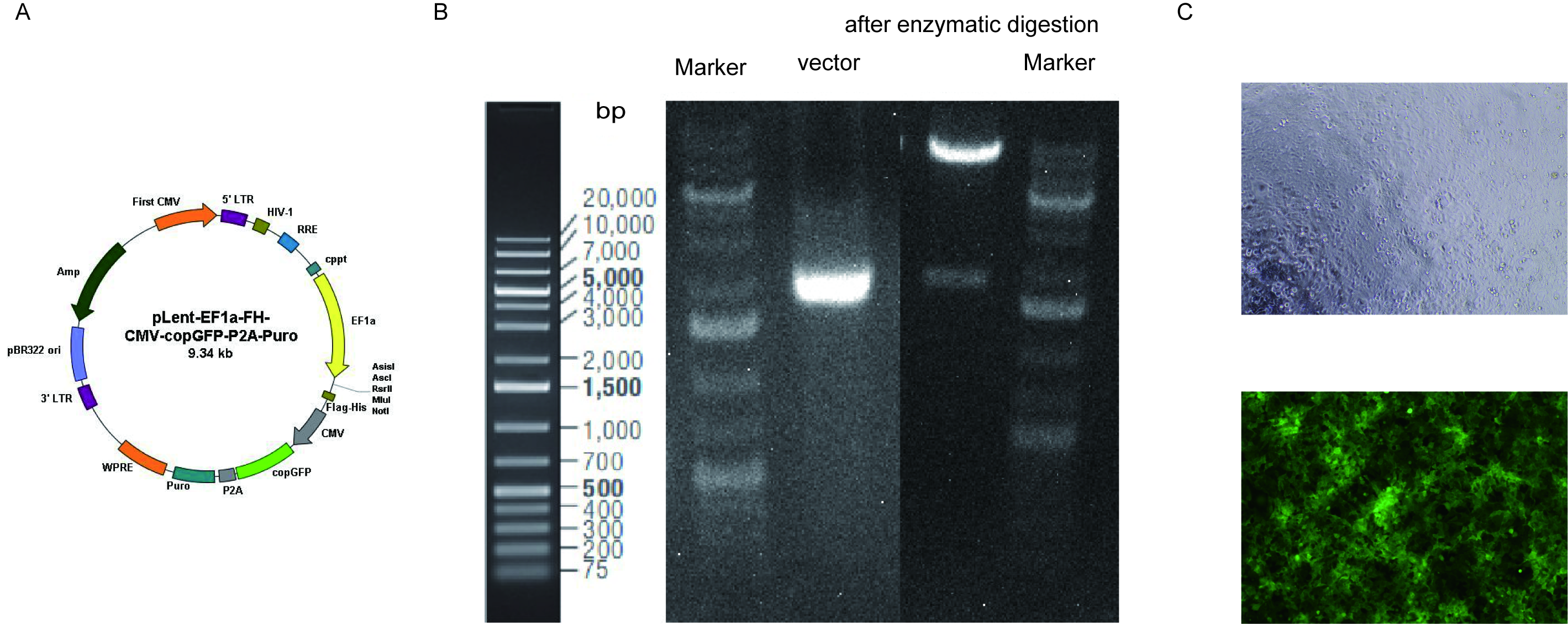

Supplement: Supplementary file 4 — Supplementary Fig. 1 [file 41419_2025_8405_MOESM4_ESM.tif]

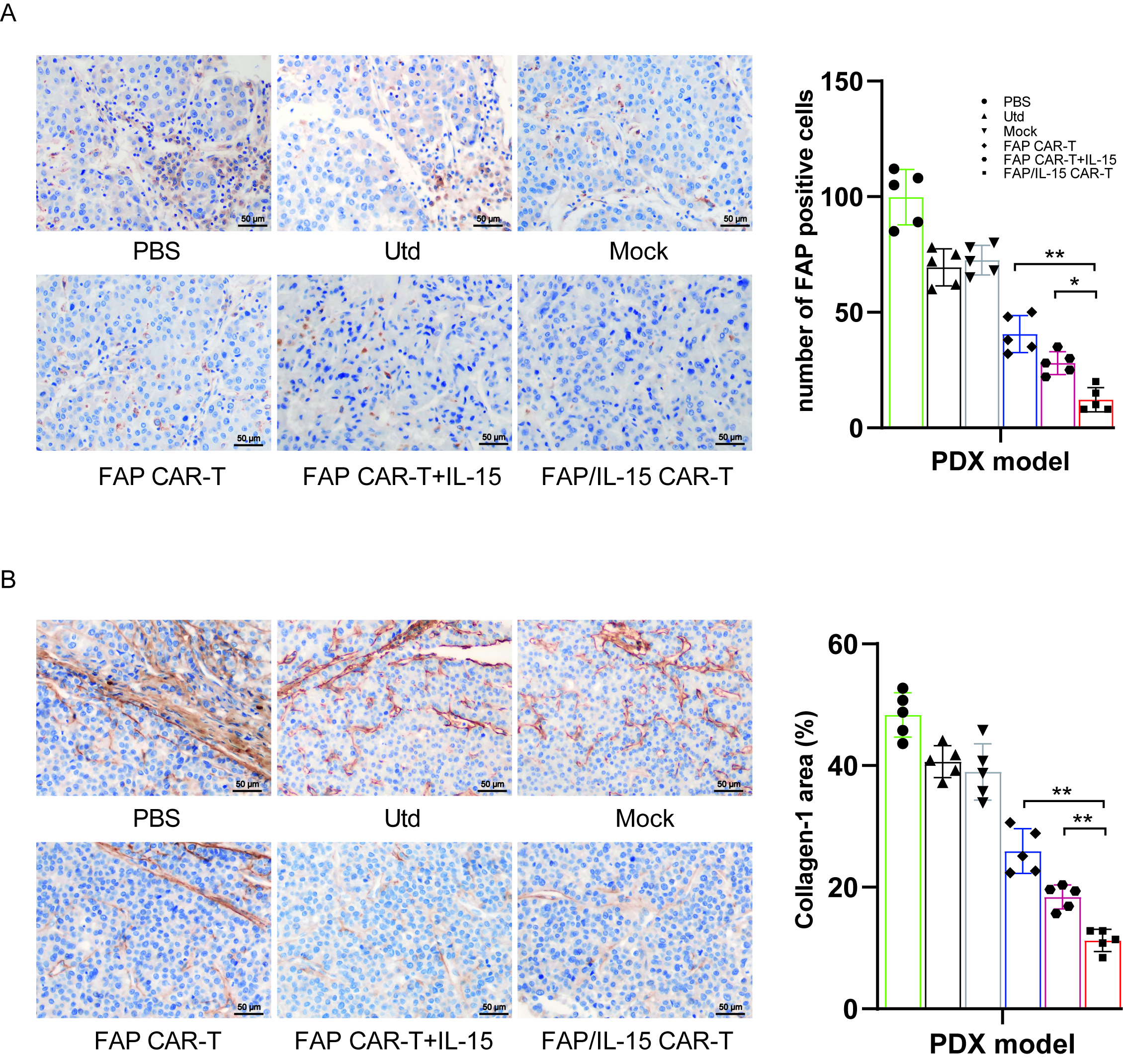

Supplement: Supplementary file 5 — Supplementary Fig. 2 [file 41419_2025_8405_MOESM5_ESM.tif]

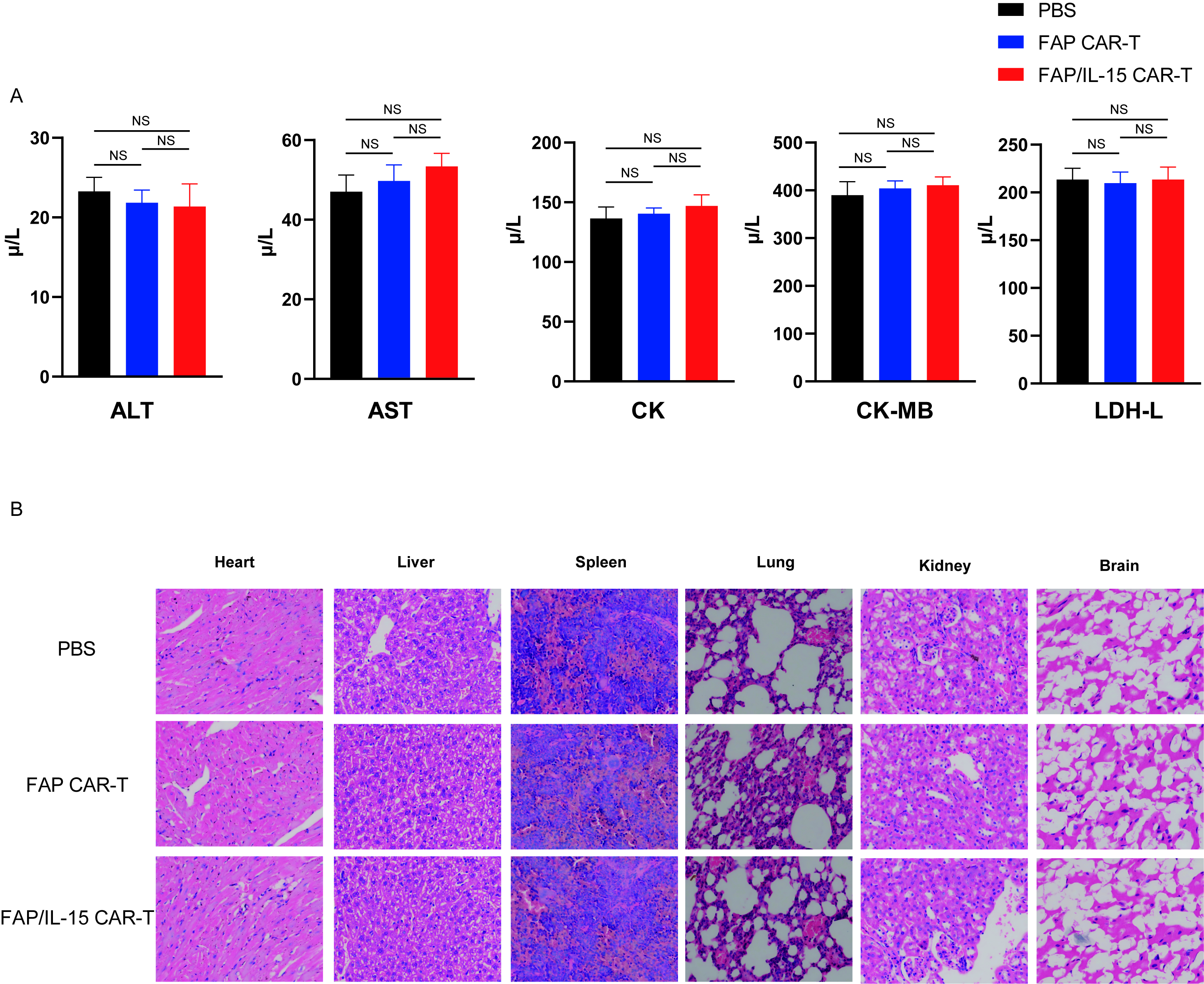

Supplement: Supplementary file 6 — Supplementary Fig. 3 [file 41419_2025_8405_MOESM6_ESM.tif]
